# Distinct patterns of distribution, community assembly and cross-domain co-occurrence of planktonic archaea in four major estuaries of China

**DOI:** 10.1186/s40793-023-00530-9

**Published:** 2023-10-07

**Authors:** Xuya Hu, Yujie Huang, Gaoke Gu, Hanjing Hu, Huizhen Yan, Huajun Zhang, Rui Zhang, Demin Zhang, Kai Wang

**Affiliations:** 1 State Key Laboratory for Managing Biotic and Chemical Threats to the Quality and Safety of Agro-products, School of Marine Sciences, Ningbo University, Ningbo, China; 2 Key Laboratory of Marine Biotechnology of Zhejiang Province, School of Marine Sciences, Ningbo University, Ningbo, China; 3 Collaborative Innovation Center for Zhejiang Marine High-Efficiency and Healthy Aquaculture, Ningbo, China; 4 Institute for Advanced Study, Shenzhen University, Shenzhen, China

**Keywords:** Planktonic archaea, Seasonality, Community assembly mechanism, Co-occurrence network, Estuarine ecosystem

## Abstract

**Background:**

Archaea are key mediators of estuarine biogeochemical cycles, but comprehensive studies comparing archaeal communities among multiple estuaries with unified experimental protocols during the same sampling periods are scarce. Here, we investigated the distribution, community assembly, and cross-domain microbial co-occurrence of archaea in surface waters across four major estuaries (Yellow River, Yangtze River, Qiantang River, and Pearl River) of China cross climatic zones (~ 1,800 km) during the winter and summer cruises.

**Results:**

The relative abundance of archaea in the prokaryotic community and archaeal community composition varied with estuaries, seasons, and stations (reflecting local environmental changes such as salinity). Archaeal communities in four estuaries were overall predominated by ammonia-oxidizing archaea (AOA) (aka. Marine Group (MG) I; primarily *Nitrosopumilus*), while the genus *Poseidonia* of *Poseidoniales* (aka. MGII) was occasionally predominant in Pearl River estuary. The cross-estuary dispersal of archaea was largely limited and the assembly mechanism of archaea varied with estuaries in the winter cruise, while selection governed archaeal assembly in all estuaries in the summer cruise. Although the majority of archaea taxa in microbial networks were peripherals and/or connectors, extensive and distinct cross-domain associations of archaea with bacteria were found across the estuaries, with AOA as the most crucial archaeal group. Furthermore, the expanded associations of MGII taxa with heterotrophic bacteria were observed, speculatively indicating the endogenous demand for co-processing high amount and diversity of organic matters in the estuarine ecosystem highly impacted by terrestrial/anthropogenic input, which is worthy of further study.

**Conclusions:**

Our results highlight the lack of common patterns in the dynamics of estuarine archaeal communities along the geographic gradient, expanding the understanding of roles of archaea in microbial networks of this highly dynamic ecosystem.

**Supplementary Information:**

The online version contains supplementary material available at 10.1186/s40793-023-00530-9.

## Background

Since recognizing the ubiquity of archaea in mesophilic marine waters from early 1990s [1, 2], the criticality of archaea in marine biogeochemical cycling has been cumulatively understood [3–5]. Under certain conditions, archaeal abundance in marginal seas or coastal waters can account for ~ 30% of the prokaryotic community [6]. However, due to the high diversity and dynamics of coastal water environments, the understanding of planktonic archaeal biogeography in typical coastal habitats is insufficient. Estuaries are typical land-to-sea transition areas [7], continuously affected by terrestrial runoffs and anthropogenic disturbances, thus often leading to multiple environmental gradients [8–10]. Understanding the distribution, community assembly, and potential microbial interactions of archaea in estuaries is crucial to expand the cognition of archaeal biogeography in coastal waters and to provide a baseline to monitor impact of regional change on the archaeal community.

The abundance and diversity of archaea in the estuarine waters could be conditionally high [11–13], and the main dominant groups are ammonia-oxidizing archaea (aka. *Thaumarchaeota* Marine Group (MG) I, now within the family *Nitrosopumilaceae* of the phylum *Thermoproteota* in the GTDB taxonomy) and heterotrophic MGII archaea (aka. *Euryarchaeota* MGII, now within the order *Poseidoniales* of the phylum *Thermoplasmatota* in the GTDB taxonomy) [14]. *Bathyarchaeota*, *Woesearchaeota*, *Nanoarchaeota*, *Thermoprofundales* (MBG-D), Asgard archaea (such as *Thorarchaeota* and *Lokiarchaeota*), and methanogens are also commonly detected in the water column [13]. Estuarine ecosystems are spatiotemporally dynamic and are often twinned with bays, and numerous studies have found that estuarine environmental gradients (such as salinity, water temperature, pH, and nutrients) largely shape the spatial distribution patterns of microbial communities in bay area [11, 12, 15, 16]. Current studies on estuarine archaeal community are mainly on single-estuary basis [12, 17–19]. For example, archaeal communities in the Pearl River estuary showed a pattern from MGI-dominated to MGII-dominated along the low-to-high salinity and nutrient gradients [20]. Several studies based on meta-analysis have provided an overview of archaeal community compositions in major estuaries around the world (including Yellow River, Yangtze River, Jiulong River, and Pearl River estuaries of China) [7, 13], expanding the understanding of distribution patterns of archaea in this typical ecosystem. However, integrating previous studies based on different sampling periods and/or sequencing protocols targeting archaea may induce interfering factors that prevent direct comparison of archaeal communities among estuaries. Correspondingly, comprehensive studies comparing planktonic archaeal communities among multiple estuaries with unified experimental protocols (including sequencing primers and platforms) during the same sampling periods are scarce. Therefore, contemporaneous and standardized multi-estuarine studies are needed to characterize distribution patterns and assembly of planktonic archaea in this typical coastal ecosystem.

In addition to the crucial role of archaea in connecting other life forms on the tree of life [21], how they interplay with bacteria and eukaryotic microbes in maintaining structure and function of the microbial food web is an emerging issue of microbial ecology. Archaea are considered as key components that interact with other microbes in complex microbiomes [22], and thus revealing their cross-domain co-occurrence patterns with other microbes is essential for understanding their positions in the microbial interactome. Recent works based on network analysis have shown some cross-domain co-occurrence patterns of archaea including temporal or spatial associations of archaea with bacteria and eukaryotic phytoplankton across various depths of the water column or regional ecological gradients of coastal waters, spatiotemporal rotation of distinct assemblages of co-occurred MGI archaea and nitrifying bacteria, common associations of MGII archaea with (photo)heterotrophic bacteria (such as SAR86 and SAR406) [23, 24]. However, the common and differential patterns of archaeal cross-domain co-occurrence relationships across multiple estuaries are largely unknown.

**Fig. 1:**
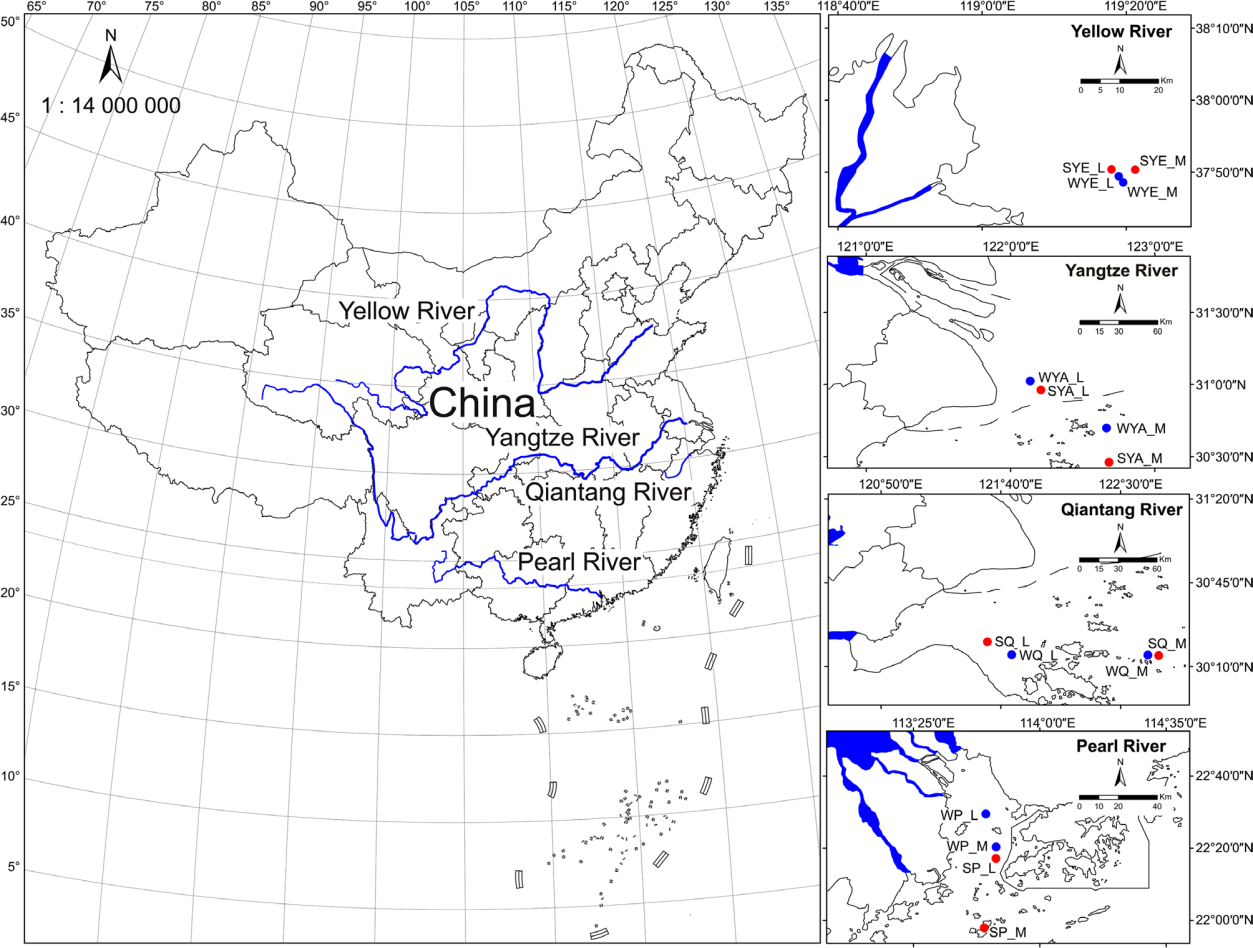
Map of the sampling stations in four major estuaries of China. The colors of symbols of stations present different seasons, Blue: Winter, Red: Summer. The meanings of the letters in the station IDs are as following: W, Winter; S, Summer; YE, Yellow River estuary; YA, Yangtze River estuary; Q, Qiantang River estuary; P, Pearl River estuary; L, Low salinity; M, Medium salinity

According to the Bulletin on the Status of Marine Ecology and Environment of China (2021), the major estuarine and/or bay ecosystems have been suffering from long-term eutrophication to varying degrees [25]. Typical ecosystems officially monitored by the Chinese government, including four major estuaries (Yellow River, Yangtze River, Qiantang River (located in the Hangzhou Bay), and Pearl River estuaries), are in a sub-healthy state. To provide comprehensive profiles of archaeal communities in this typical ecosystem highly disturbed by terrestrial and/or anthropogenic sources, we selected the four estuaries mentioned above from north to south along the coastline of China across climatic zones (~1,800 km) (Fig. 1). Surface waters along the ecological gradient of each estuary were collected during a winter cruise and a summer one. Using 16S rRNA gene amplicon sequencing combined with quantitative ecological models and microbial network construction, we found: (1) distinct distribution patterns of archaea across the estuaries regardless of season; (2) distinct community assembly mechanisms of archaea across the estuaries; (3) contrasting cross-domain microbial co-occurrence patterns of archaea across the estuaries, collectively highlighting the lack of common patterns in the dynamics of estuarine planktonic archaeal communities along the geographic gradient.

## Materials and methods

### Study sites, sampling, and physicochemical analyses of waters in the estuaries

Four major estuaries of China along a latitude gradient, including Yellow River, Yangtze River, Qiantang River, and Pearl River estuaries, were investigated during the winter and summer cruises. Three of them are the estuaries of the big four rivers in China (including Yellow River, Yangtze River, and Pearl River), and Qiantang River estuary is located in the Hangzhou Bay as one of the largest and most developed bay areas in China. Two sampling stations were set in each estuary according to the salinity level (low (~10 psu) and medium (~25 psu)). Due to the seasonality in environmental conditions including water temperature (ranging − 0.1–18.4 ^o^C and 24.6–29.7 ^o^C in the winter and summer cruises, respectively; Fig. S1), the sampled stations were somewhat different in the two cruises (winter: January 2016; summer: August-September 2016) (Fig. 1). The surface waters (at ~0.5-m depth) were collected at each station with five biological replicates, and a total of 80 samples were collected. The water samples were prefiltered through a 100-mesh (~150-µm) sterile nylon mesh and then filtered onto a 0.2-µm polycarbonate membrane (Millipore, USA). The filters were stored with dry ice on board and then at -80 °C after being transported back to the laboratory. Water temperature, pH, dissolved oxygen (DO), and salinity were measured on board using a probe (YSI550A, USA). Nitrate, nitrite, phosphate, silicate, and chemical oxygen demand (COD) were determined according to the standard methods [26].

### DNA extraction, 16S rRNA gene amplification, and Illumina sequencing

Total DNA on the filters was extracted using a Power Soil DNA Isolation Kit (MOBIO, USA). To study archaea and their co-occurrence with bacteria from a perspective of the whole prokaryotic community, the V4 region of 16S rRNA genes was amplified using a archaeal-bacterial universal primer set 515FY (5’-GTGYCAGCMGCCGCGGTAA-3’) and 806RB (5’-GGACTACNVGGGTWTCTAAT-3’) with dual barcodes [27, 28]. The coverage of the primers for both archaea and bacteria has been evaluated and discussed in our previous work [29]. An amount of 10 ng purified DNA template from each sample was amplified in triplicate with a 20-µl reaction system under the following conditions: initial denaturation at 95 °C for 3 min; then 28 cycles of denaturation at 95 °C for 30 s, annealing at 55 °C for 30 s, and extension at 72 °C for 45 s; with a final extension at 72 °C for 10 min. Triplicate PCR products for each sample were pooled and purified using a PCR fragment purification kit (TaKaRa, Japan), quantified using a Quant-It Pico Green kit with a Qubit fluorometer (Life Technologies, USA), and sequenced on an Illumina MiSeq machine (Illumina, USA).

### Sequence processing

Paired reads were joined with FLASH using default settings [30]. The joined reads were processed using USEARCH (v11.0.667) [31]. Briefly, the joined sequences were quality-checked (maximum expected errors (maxEE) = 1.0) and de-replicated using the script *usearch11 -fastx_uniques*. Subsequently, de-noise, chimera-check, and identification of ZOTUs (Zero-radius Operational Taxonomic Units, also known as ASVs (Amplicon Sequence Variants)) were processed using UNOISE3 algorithm [32] with the script *usearch11 -unoise3* (minisize = 8). A total of 17,277 ZOTUs were yielded. To obtain ZOTU abundances, original joined reads were mapped to ZOTU sequences at 100% similarity using the script *usearch11 -otutab*. The ZOTUs were taxonomically assigned against the SILVA_138_SSURef_Nr99 reference database [33] using Blastn v2.13.0+, with a percentage of identity > 75%, max_target_seqs = 1, and an *e*-value < 0.0001. To further improve the taxonomic annotation of archaeal ZOTUs, they were also assigned against Genome Taxonomy Database (GTDB) [34]. Sequences that cannot be assigned to bacteria or archaea, as well as chloroplast and mitochondrial sequences were removed. The full prokaryotic dataset (n = 80) yield 16,546 ZOTUs comprising 2,387,656 qualified reads (read range 10,633 − 54,063, mean = 29,846 per sample). Relative abundances of major archaeal groups in prokaryotic communities and neutral model fitting of prokaryotic communities were calculated based on a prokaryotic ZOTU table rarefied at 10,600 reads per sample. The archaeal ZOTU table was separated from the unrarefied prokaryotic ZOTU table, and the archaeal sub-dataset (n = 80) yield 540 ZOTUs comprising 185,619 qualified reads (read range 4–14,742, mean = 2,320 per sample). To deal with very uneven archaeal sequence numbers across samples, archaeal ZOTU abundances (after discarding samples with < 100 reads) were normalized by cumulative sum scaling transformation [35] for principal coordinate analysis (PCoA) and permutational multivariate analysis of variance (PERMANOVA) that characterize the compositional variation of archaeal communities. Three of the samples from each station in Yellow River estuary during the winter cruise (hereinafter referred to as WYE_L and WYE_M samples) and all five samples from the low salinity station in Peal River estuary during the winter cruise (WP_L samples) were excluded prior to these analyses, due to their low read counts.

### General statistical analyses

The map of sampling stations and geostatistical analyses were performed using ArcGIS 10.4. Three-way repeated measures analysis of variance (ANOVA) was applied to test the influence of estuary, season, station, and their interaction on the relative abundances of total archaea and dominant archaeal taxa in prokaryotic communities using SPSS Statistics 26. Heatmap was used to visualize Spearman’s correlation of environmental and geographic factors with the relative abundance of total archaea and major archaeal genera in prokaryotic communities during the winter or summer cruise using the R package “ggplot2” [36]. Principal coordinate analysis (PCoA) based on Bray-Curtis dissimilarity was used to visualize the compositional variation total archaeal communities across estuaries, seasons, and stations. Permutational multivariate analysis of variance (PERMANOVA) was used to test the influence of estuary, season, station, and their interactions on the variation in archaeal community composition. The effects of geographic distance, environmental variables (based on Euclidean distance), and bacterial community composition (based on Bray-Curtis dissimilarity) on archaeal community composition were explored with (partial-)Mantel tests and corresponding tests were also done for bacterial community composition. These analyses were performed using the R package “vegan” unless otherwise indicated [37].

### Inferring the assembly processes of archaeal taxa

Sloan neutral model [38] was used to infer ecological processes governing the assembly of archaeal taxa into the prokaryotic community. Fitting of the neutral models was performed for prokaryotic communities in each estuary in the winter or summer cruise with the metacommunity cross all four estuaries as the source (aka. species pool) using the R scripts previously reported [39]. The goodness of model fitting was evaluated by R^2^. The estimated migration rate (m), as a measure of dispersal limitation, was calculated using the R package “minpack.lm” [40], and higher m values indicate less dispersal-limited. The 95% confidence interval around the model prediction were evaluated using the R package “Hmisc” [41]. The ZOTUs within the confidence intervals were considered as neutrally distributed, suggesting that they were assembled into the local communities by stochastic dispersal and ecological drift. The ZOTUs above the upper boundary of the confidence interval hold strong probability of preference for certain local conditions, thus being selected for, while the ZOTUs below the lower boundary of the confidence interval are underrepresented in certain local conditions compared to the model prediction by their abundance in the source, suggesting being selected against and/or being dispersal-limited from the source [42]. Cumulative relative abundances of neutrally-distributed and non-neutrally distributed archaeal ZOTUs were calculated to infer the relative importance of neutral (stochastic) and selection (deterministic) processes on governing archaeal community assembly in each estuary. The Venn diagram was applied to analyze the numbers of archaeal ZOTUs being unique and shared in the four estuaries during the winter or summer cruise.

### Association network analyses

To discover the across-domain co-occurrences of archaea with bacteria and eukaryotic phytoplankton in each estuary, bacteria and chloroplast reads were retained in the unrarefied ZOTUs tables for network analyses. Chloroplast 16S rRNA genes are often used to profile photosynthetic protists (eukaryotic phytoplankton) [23, 43], facilitating assessment of the dynamics of photosynthetic protists without the uncertainties of distinguishing heterotrophic and autotrophic protists with the 18S rRNA genes while with less variability of gene copy numbers between taxa than for the 18S rRNA genes [43]. The taxonomy of chloroplast ZOTUs were further assigned against PhytoRef database [44] using Blastn. To reduce compositional effects and spurious associations, ZOTUs with sequence abundance ≥ 100 reads and appearing in ≥ 15% of samples were retained for network calculations [45, 46].

Eight sub ZOTUs tables were generated corresponding to each estuary in both seasons, and microbial associations combining environmental factors were inferred using FlashWeave (sensitive = true, heterogeneous = false, alpha = 0.001, n_obs_min = 10, normalize = true) [47]. FlashWeave was used because of its merits on detecting and removing indirect (i.e., purely correlational) associations to construct direct association networks based on local-to-global learning (LGL) approach proposed by Aliferis et al. [48], a constraint-based causal inference framework for the prediction of direct relationships between variables, thus reducing false or suspicious associations. It furthermore allows the seamless integration of environmental factors, to estimate their influence on microbial associations and then to remove indirect associations driven by them. Given that FlashWeave does not support missing environmental data, the environmental factors integrated here include water temperature, salinity, and pH. The direct associations are considered as robust and visualized using Chord diagrams with the R package “circlize” [49]. The topological features of the networks were then calculated with Gephi [50]. The bubble plot was created to summarize the cross-domain associations between archaea and bacteria using the R package “ggplot2” [36]. The topological role of each node in the networks was estimated based on its within-module connectivity (*Zi*) and among-module connectivity (*Pi*) [51] using the R package “igraph” [52]. According to the suggested *Zi* and *Pi* degree threshold [53], all nodes were categorized into four subcategories: peripherals (nodes connected in modules with few outside connections, *Zi* ≤ 2.5 and 0 ≤ *Pi* ≤ 0.62), connectors (nodes that connect modules, *Zi* ≤ 2.5 and *Pi* > 0.62), module hubs (highly connected nodes within modules, *Zi* > 2.5 and *Pi* ≤ 0.62), and network hubs (highly connected nodes within entire network, *Zi* > 2.5 and *Pi* > 0.62).

## Results and discussion

### Variability of environmental conditions across the estuaries

In general, water temperature across the estuaries followed a typical low-to-high gradient along the high-to-low latitude gradient (with similar levels in Yangtze River and Qiantang River estuaries) during the winter cruise, while similar levels across four estuaries were found during the summer cruise (Fig. S1). Yellow River estuary had the highest level of DO, with a high-to-low gradient along the high-to-low latitude gradient during the winter cruise. Most environmental variables showed seasonal and spatial (both intra- and inter-estuary) variability to varying degrees. There were overall similar environmental conditions in Yangtze River and Qiantang River estuaries because of their close geographic proximity. In addition, significant differences in other variables, including pH, DO, and nutrients (NO_2_^−^, NO_3_^−^, SiO_3_^2−^, and PO_4_^3−^), were commonly found between stations with low (~10 psu) and medium (~25 psu) salinity levels in each estuary, confirming the multiple co-gradients with salinity.

### Distinct distribution patterns of archaea across the estuaries

**Fig. 2:**
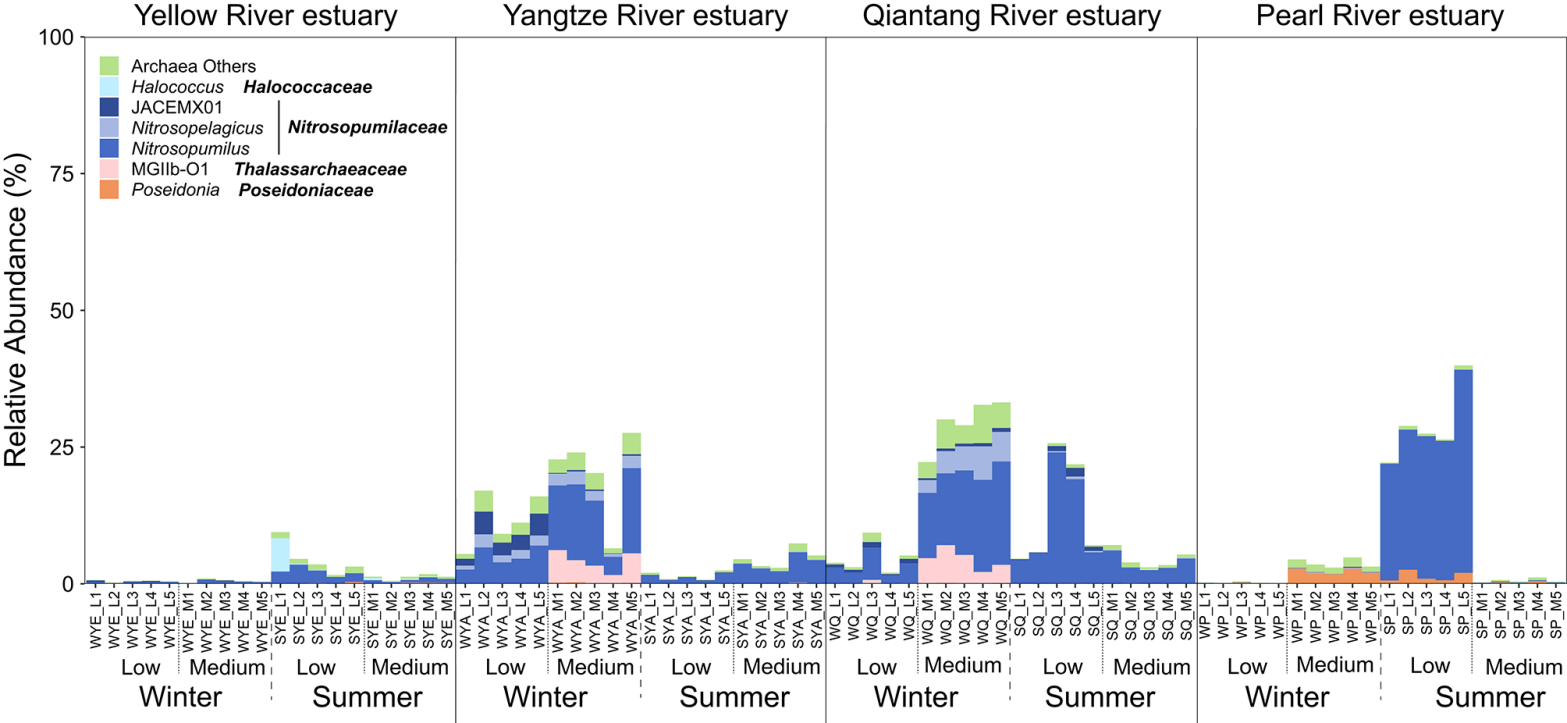
Relative abundances of major archaeal genera (with relative abundance ≥ 1% in at least one sample and average relative abundance ≥ 0.1% in all samples) in prokaryotic communities in four major estuaries across seasons and stations. Bold taxon names are the families the genera affiliated to. Low and medium present stations with corresponding salinity level. Refer to Fig. 1 for the meanings of the letters in the station IDs

Coastal marine ecosystems, especially estuaries are diversified by varying environmental conditions (such as those mentioned above) and distinct types and strength of anthropogenic disturbances [10, 13, 18, 54], which leads to the difficulty in capturing common patterns in niche partitioning of archaea. Therefore, simultaneous survey of major representative estuaries along the geographic gradient could provide insights into common and distinct patterns of archaeal community in this typical coastal ecosystem. In this study, the relative abundance of archaea in the prokaryotic community of surface waters drastically varied across estuaries (*P* < 0.001) and was significantly influenced by the interactions of *Estuary*, *Season*, and *Station* (all *P* < 0.001; Fig. 2 and Table S1). Seasonality was more influential than station in determining the relative abundance of archaea in each estuary (Table S1). Yellow River estuary had constantly low relative abundance of archaea (1.7% in average, ranging 0.02–9.5%) regardless of season or station, while Qiantang River (12.9% in average, ranging 2.1–33.2%) and Yangtze River estuaries (9.5% in average, ranging 0.8–27.6%) had relatively higher archaeal abundance (Fig. 2). The relative abundance of archaea in Pearl River estuary (8.4% in average, ranging 0.06–39.9%) showed the most dramatic seasonality and station-dependency cross the estuaries (Fig. 2 and Table S1). These results reflect the importance of unveiling the distribution pattern of archaea in the context of the whole prokaryotic community, suggesting the ebb and flow between archaea and bacteria in the estuarine surface waters are highly dynamic.

Corresponding to a review meta-analyzing estuarine planktonic archaea at the global scale [7], archaeal community in most samples of Yellow River, Yangtze River, and Qiantang River estuaries were predominated by ammonia-oxidizing archaea (AOA)/MGI (*Nitrosopumilaceae*, 74.3% in average, primarily *Nitrosopumilus*, accounting for 60.3%), while in Pearl River estuary, *Nitrosopumilus* only dominated the archaeal community in the low-salinity station in the summer cruise (93.6% in average) (Fig. 2). The relative abundance of *Nitrosopelagicus* was also considerable in Yangtze River estuary and the medium-salinity station of Qiantang River estuary in the winter cruise (10.6% and 15.2% in average, respectively). *Nitrosopelagicus*-like AOA are the major population in open ocean surface waters globally [55], but their distribution pattern and corresponding determinants in coastal/estuarine ecosystem are unclear. It seems that the bloom of *Nitrosopelagicus* in these two estuaries was highly season- and salinity-dependent. MGII archaea are ubiquitous in marine waters, and the vertical and horizontal niche partitioning between MGII and MGI were respectively found along the water columns in the open ocean [3, 23, 56] and across coastal surface waters at the regional scale [57]. However, the niche partitioning among MGII archaea at the finer taxonomic scales in estuarine/coastal water remain largely unknown. Among the MGII genera, MGIIb-O1 was only dominant in the medium-salinity stations of Yangtze River and Qiantang River estuaries in the winter cruise (20.0% and 15.0% in average, respectively), while *Poseidonia* was occasionally dominant in Pearl River estuary across stations and seasons. This suggests that both MGII genera tend to be more abundant in stations with higher salinity in the winter, with a clear estuary-specific niche partitioning. Also, the *Poseidoniaceae* (MGIIa) genus *Poseidonia* showed considerable abundance in Pearl River estuary in the summer cruise (especially in the low-salinity station; 4.7% in average), likely due to its preference for warmer waters as compared to *Thalassarchaeaceae* (MGIIb) taxa [58]. However, MGIIb could also be dominant in coastal waters during warm months [59]. In addition, *Halococcus* (affiliated to *Halobacteriota*) was only considerable in a single summer sample from the medium-salinity station of Yellow River estuary (Fig. 2). Many previous studies reported the associations of multiple environmental factors (like salinity, water temperature, inorganic nutrients, and DO) with distribution patterns of major archaeal groups [13, 57, 60]. We also found common associations between the relative abundance of total archaea and dominant archaeal genera and environmental factors; however, strong distinctions in terms of the combination of correlative factors and/or direction of correlation coefficients of the given factors were observed between the two cruises (Fig. S2). This inconsistency may be due to the highly dynamic environmental conditions in the estuaries shaped by complex interactions of geographic location, season, and terrestrial input via river runoff, on the other hand, indicating potential stochasticity in archaeal community assembly as suggested by previous studies [57, 29]. Due to the limited seasons and stations we monitored, this study mainly focused on comparing archaeal communities among estuaries in each cruise to avoid overstating or simplifying seasonal patterns, which are worthy of future investigation by performing a sampling scheme with higher spatiotemporal coverage to reveal extensive seasonal changes.

**Fig. 3:**
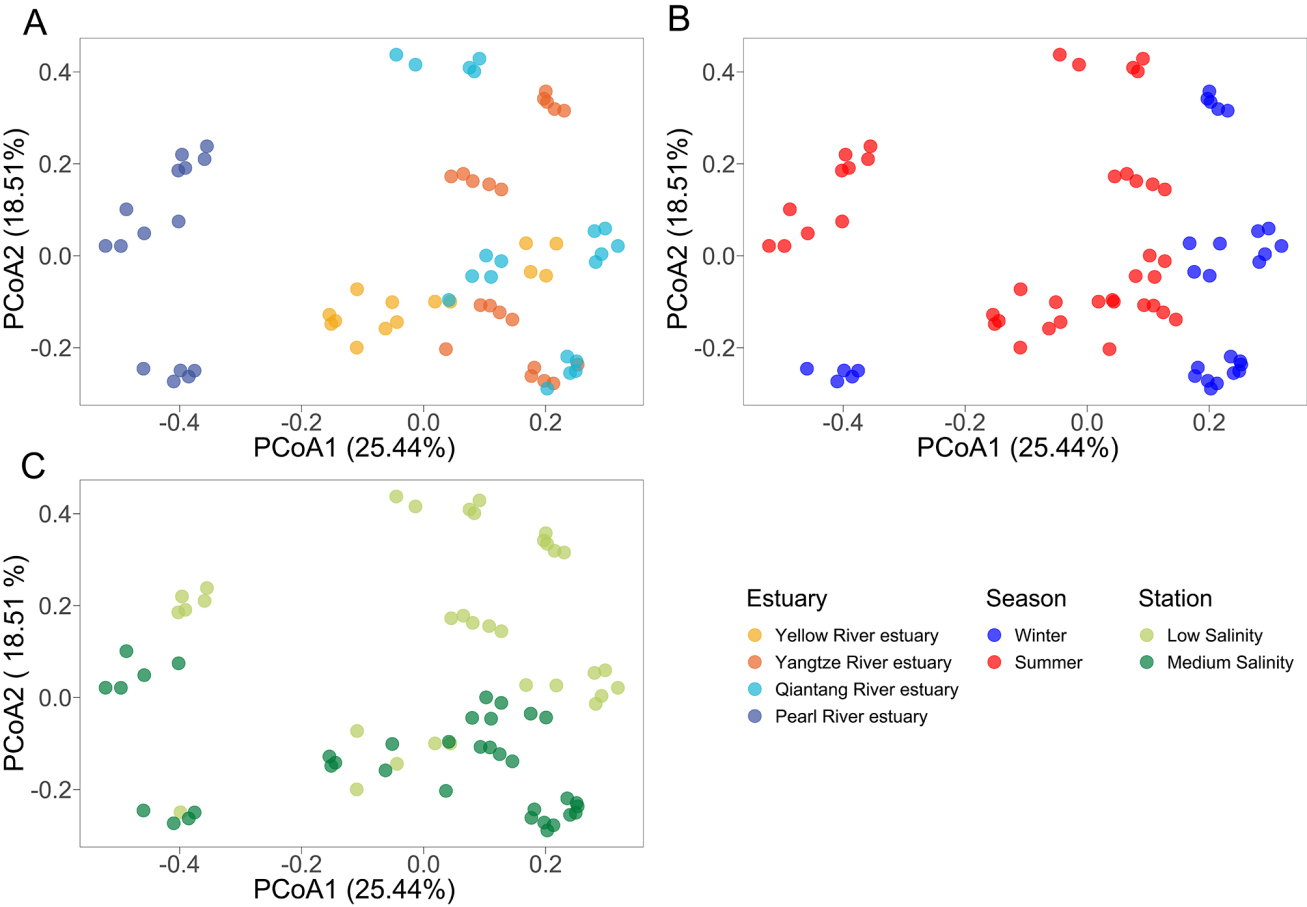
Principal coordinate analysis (PCoA) based on Bray-Curtis dissimilarity showing the variation in archaeal community composition. The plots were color-mapped by estuary (A), season (B), and station (C), respectively. Three WYE_L samples, three WYE_M samples, and all WP_L samples were excluded due to low read counts. Refer to Fig. 1 for the meanings of the letters in above station IDs

The compositions of archaeal communities varied with *Estuary*, *Season*, and *Station* (indicating salinity and other co-gradients including NO_3_^−^, SiO_3_^2−^, and COD) (Fig. 3). *Estuary* was the most influential factor shaping archaeal community composition (R^2^_PERMANOVA_ = 0.367, *P* = 0.001), followed by *Station* and *Season*, and the interactions of any two of them or all of them also showed significant effects (all *P* = 0.001) (Table 1). On the single-estuary basis, seasonality was more important than station-dependent pattern in archaeal community composition in Yellow River (where the *Station* effect was not significant) and Pearl River estuaries, while Yangtze River and Qiantang River estuaries exhibited the opposite trend. Mantel tests showed that bacterial community composition was the strongest driver of compositional variation of archaeal community in the winter cruise (ρ = 0.880, *P* = 0.001; Table S2) even after controlled by other key drivers including geographic distance and abiotic environmental factors such as water temperature, DO, COD, SiO_3_^2−^, and NO_3_^−^ (all ρ > 0.6, *P* = 0.001), but only geographic distance (ρ = 0.628, *P* = 0.001) and water temperature (ρ = 0.374, *P* = 0.001) remained correlative after controlled by bacteria community composition. In the summer cruise, geographic distance was the strongest determinant of archaeal community composition (ρ = 0.700, *P* = 0.001), and bacterial community composition also showed a strong effect (ρ = 0.597, *P* = 0.001). But NO_2_^−^ as the only abiotic environmental factor with a considerable effect (ρ = 0.499, *P* = 0.001) turned insignificant after controlled by bacterial community composition (ρ = -0.046, *P* = 0.754). It is worth noting that the bacterial community composition was more constrained by environmental divers after controlled by archaeal community than for environmental constraints to archaeal community controlled by bacterial community (Tables S2-S3). This suggests that bacterial community composition was likely more important in shaping archaeal community than for archaea in shaping bacterial community, via their interactions as indicated by the common archaea-bacteria associations presented below. Also, the key role of geographic distance in shaping archaeal community in both cruises was corresponding to the estuary-dependent patterns. This explains the relatively similar compositions of archaea in Yangtze River and Qiantang River estuaries with geographic proximity, due to which the plume of Yangtze River through the estuary can largely influence the environmental conditions in Qiantang River estuary [61]. The environmental determinants of archaeal community composition identified in either of the cruises, such as water temperature, DO, salinity, and SiO_3_^2−^, were consistent with many previous reports [20, 23, 57]. Although some strong environmental drivers of compositional variation of archaeal community were identified in the winter cruise (despite some of them likely affected archaeal community via governing bacteria), key environmental drivers remain a question for the summer cruise. Therefore, future efforts should be made to collect typical and comprehensive environmental profiles (including organic matters, sulfate, etc.) for unveiling the dynamics in environmental determinants of estuarine archaeal community. Collectively, we confirmed distinct distribution patterns of archaea across four major estuaries along the geographic gradient in terms of dominant groups and community compositions.

**Table 1:** Permutational multivariate analysis of variance (PERMANOVA) based on Bray-Curtis dissimilarity to quantify the effects of estuary, season, station, and their interactions on compositional variation of archaeal communities. Since all WP_L (the samples of low salinity in Pearl River estuary during the winter cruise) samples were removed due to low archaeal read counts, the interaction of season and station in Pearl River estuary cannot be tested

Estuary	Factor	R^2^	*P*
All	*Estuary*	0.367	**0.001**
	*Station*	0.115	**0.001**
	*Season*	0.105	**0.001**
	*Estuary × Season*	0.100	**0.001**
	*Estuary × Station*	0.085	**0.001**
	*Station × Season*	0.019	**0.001**
	*Estuary × Station × Season*	0.037	**0.001**
Yellow River estuary	*Station*	0.092	0.065
	*Season*	0.432	**0.001**
	*Station × Season*	0.046	0.357
Yangtze River estuary	*Station*	0.412	**0.001**
	*Season*	0.255	**0.001**
	*Station × Season*	0.122	**0.001**
Qiantang River estuary	*Station*	0.331	**0.001**
	*Season*	0.294	**0.001**
	*Station × Season*	0.143	**0.001**
Pearl River estuary	*Station*	0.318	**0.001**
	*Season*	0.421	**0.001**

Data in bold indicate significance (*P* < 0.05). R^2^ values represent the proportion of variance constrained by factors or their interactions

### Assembly processes of archaeal taxa into the prokaryotic community

The assembly of archaea in coastal waters were previously reported to be more stochastically governed relative to determinism at the regional scale, which is contrasting to that of bacteria [57, 29]. A study focusing on 21 China’s coastal wetlands also demonstrated that stochastic processes regulated the assembly of planktonic archaea [62]. However, the variability and/or consistency in assembly mechanisms of archaea in multiple estuaries at different climatic zones is unclear. In this study, we found that archaeal community compositions showed much weaker association with water environmental factors compared with geographic distance, and thus unveiling the assembly mechanism of archaea is crucial to explain how the estuary-specificity of archaeal compositions formed. We evaluated the assembly mechanisms of archaea in terms of how archaeal taxa from all-estuary- or local-estuary-scale pool were assembled into local prokaryotic communities in each estuary. When assuming the prokaryotes across all the four estuaries (all-estuary-scale pool) as the source community, the assembly of prokaryotic communities did not fit the neutral model in almost all the estuaries (R^2^ < 0, except in Yangtze River estuary in the summer cruise with R^2^ = 0.191; Fig. S3), and the numbers of shared archaeal ZOTUs across the four estuaries were only 22 and 30 in the winter and summer cruises, respectively (accounting for 4.85% and 7.21% of ZOTUs, respectively; Fig. S4), suggesting that cross-estuary dispersal of archaea were largely limited and/or archaeal communities in each estuary were likely selected by local environmental conditions. The greatest number of shared archaeal ZOTUs were found between Yangtze River and Qiantang River estuaries (accounting for 86.4% and 60.6% of ZOTUs across estuaries in the winter and summer cruises, respectively), again emphasizing the effect of geographic proximity between these two estuaries.

**Fig. 4:**
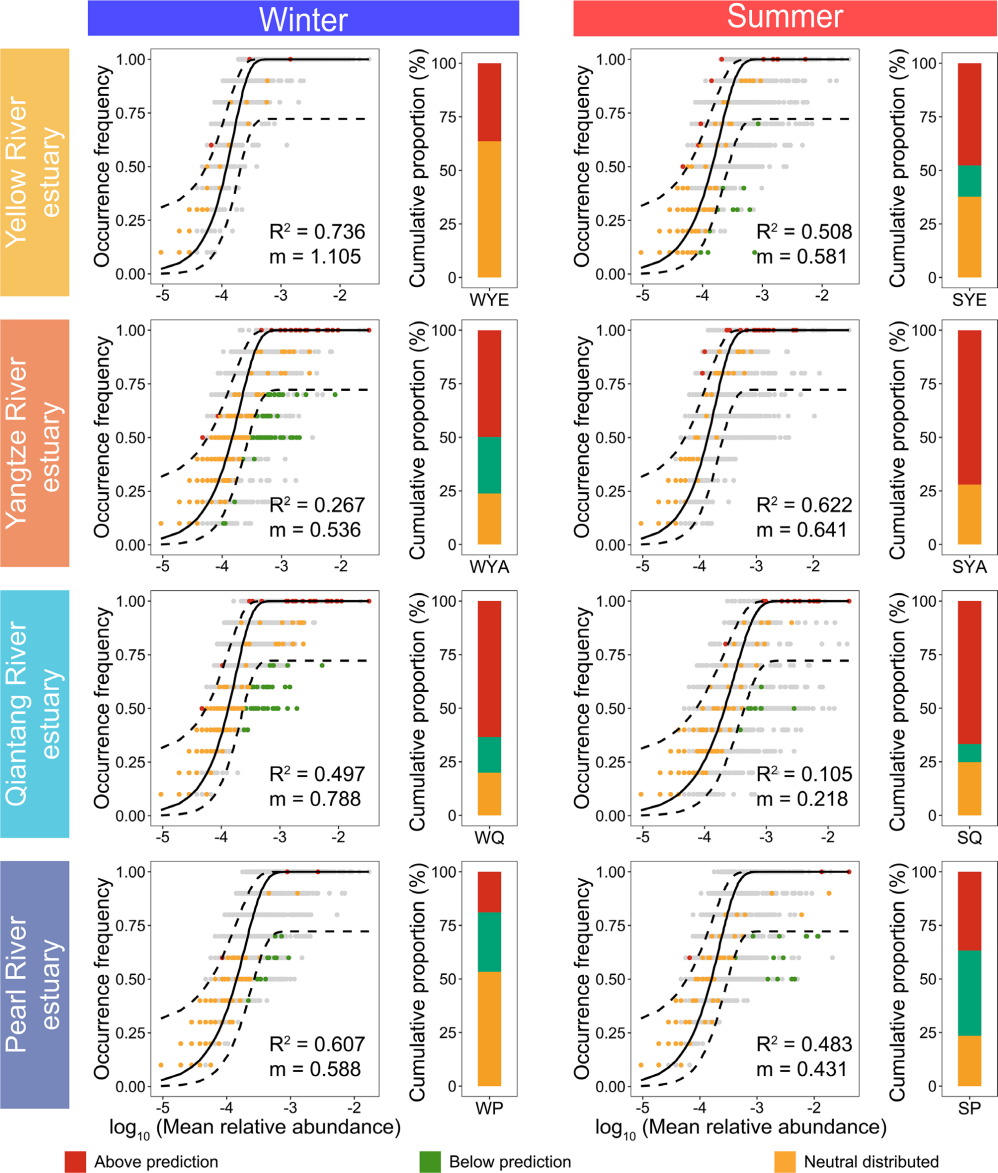
Fit of the neutral models for prokaryotic communities in each estuary during the winter or summer cruise with the metacommunity in each estuary as the source. Archaeal ZOTUs that occurred more frequently than the model prediction are shown in red, while those occurred less frequently than predicted are shown in green. Dashed lines represent 95% confidence intervals around the model prediction and the archaeal ZOTUs fall within the confidence intervals are considered as neutrally distributed and shown in orange. Bacterial ZOTUs are shown in grey. R^2^ values present the goodness of model fitting, ranging from 0 (no fit) to 1 (perfect fit). The histograms show cumulative relative abundances of three categories of archaeal ZOTUs.

When assuming the prokaryotes in each estuary as the source community, the occurrence frequency of prokaryotic ZOTUs fit the neutral model regardless of estuaries or seasons (but with various fitting goodness: R^2^ = 0.105 ~ 0.736; Fig. 4). Three categories of archaeal ZOTUs were marked, whose cumulative relative abundances showed estuary-dependency in assembly mechanism of archaea (Fig. 4). In the winter cruise, archaeal assembly in Yellow River estuary was dominantly governed by neutral (stochastic) process (accounting for 63.6% of cumulative abundance), while selection (deterministic) processes similarly dominated the assembly of archaea in Yangtze River and Qiantang River estuaries (accounting for 76.2% and 80.1% of cumulative abundance, respectively). Archaeal assembly in Pearl River estuary was nearly balancedly governed by stochastic and deterministic processes (53.3% versus 46.7% of cumulative abundance). These results suggest that assembly mechanisms of archaea varied with estuaries. However, in the summer cruise, selection processes predominated archaeal community assembly in all the four estuaries with differences in relative importance of selection for and selection against local environmental conditions, indicating that the variability or consistency in assembly mechanism of estuarine archaea could switch with season. Our previous work has revealed seasonal variability of the relative importance of deterministic and stochastic assembly of bacteria, while seasonal consistency of stochasticity-dominated assembly of archaea across coastal waters at the regional scale [29]. Our current work generally suggests the power of local environmental conditions in selecting archaeal communities in each estuary. But in Yellow River estuary with extremely strong mixing of water masses, stochasticity-dominated mechanism occurred seasonally. On the other hand, the much lower relative abundance of archaea in Yellow River estuary compared with that of the other estuaries suggests a smaller population size of archaea, which is more susceptible to ecological drift (as a major stochastic process caused by random birth and death of individuals) [63].

### The role of archaea in microbial networks and their associations with other microbes across the estuaries

**Fig. 5:**
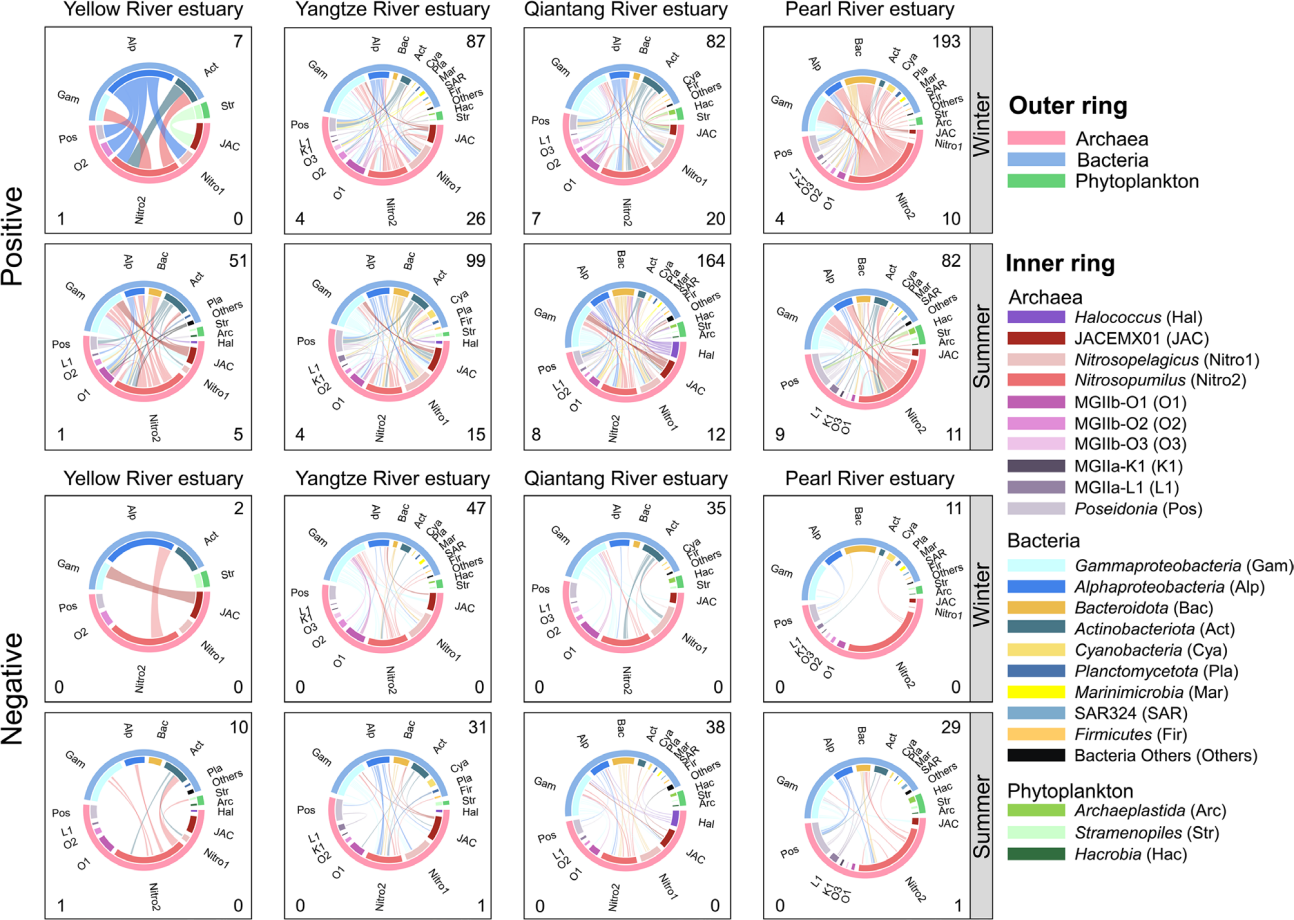
Chord diagrams showing the direct archaeal intra-domain associations and cross-domain associations with bacteria and eukaryotic phytoplankton. The numbers in upper right, bottom left, and bottom right corners of each panel present the numbers of archaea-bacteria, archaea-phytoplankton, and archaea-archaea edges, respectively

With the expanding knowledge about archaeal diversity [3, 64], the role of them in complex microbial communities and how they function in microbial food web has attracted broad attention [65, 66]. Although network analyses were extensively used to describe co-occurrence patterns of microbes [23, 24, 67], the role of archaea in planktonic microbial network is less known. Previous studies have reported co-occurrences between MGI archaea and nitrifying bacteria (including *Nitrospina* and/or *Nitrospira*), MGII archaea and (photo)heterotrophic bacteria, MGII archaea and eukaryotic phytoplankton [23, 57, 43, 68]. But how the cross-domain associations of archaea vary with estuaries is still an open question. In this study, we used a local-to-global learning framework to infer direct microbial associations. Overall, archaeal-related co-occurrence networks showed certain differences in size and topology across four estuaries in two seasonal cruises (Fig. 5 and Table S4). Positive associations dominated co-occurrence relationships in all the archaeal-related networks (81.41% in average, ranging 71.34–94.95%) with intra- and cross-domain associations of archaea dominated by positive edges (98.02% and 79.74% in average; Table S4). The dominance of positive archaea-related edges was also observed across a large scale of coastal wetlands [62], suggesting potential mutualistic relationships between archaea and other microbes in coastal waters. According to the connectivity of network nodes, most archaeal ZOTUs were peripherals and/or connectors in the microbial networks regardless of estuaries or cruises (Fig. S5). Several *Nitrosopumilus* and *Halococcus* ZOTUs served as module hubs in Pearl River/Qiantang River estuary, but no archaeal ZOTUs served as network hubs. This suggests that archaea did not likely serve as keystone taxa in the microbial networks, though broad cross-domain associations of archaea were detected. While, the case of archaea being keystones in planktonic microbial networks have been found in the coastal wetlands [62] and Monterey Bay [24]. These inconsistent observations suggest the role of archaea in microbial networks could vary with ecosystems.

**Fig. 6:**
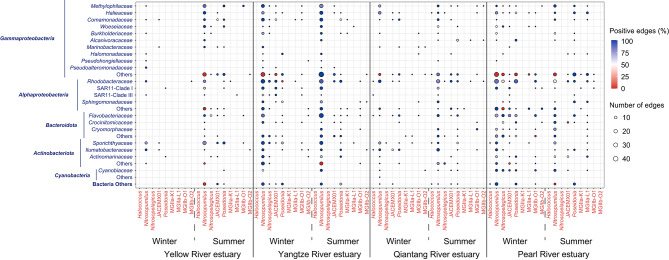
Bubble plot summarizing the direct archaea-bacteria associations in each estuary during the winter and summer cruises. Only the bacterial families with more than 4% edges of archaea-bacteria edges in each estuary during the winter or summer cruise are shown. The size of bubbles indicates the number of edges. The color of bubbles indicates the percentages of positive edges. Archaeal and bacterial taxa are shown in red and blue fonts, respectively

Archaea-related associations mainly involved 10 genera affiliated to four families including *Nitrosopumilaceae*, *Poseidoniaceae* (MGIIa), *Thalassarchaeaceae* (MGIIb), and *Halococcaceae* (Fig. 5). Most intra-domain associations of archaea occurred among *Nitrosopumilaceae* taxa. The *Nitrosopumilaceae* genera *Nitrosopumilus*, *Nitrosopelagicus*, and JACEMX01 generally had more across-domain associations than other archaeal genera did. Interestingly, we found the relative abundance of a given archaeal genus not necessarily associate with its cross-domain connectivity in the network (Fig. S6). For example, *Nitrosopumilus* with the highest relative abundance did possess the highest across-domain connectivity (as estimated by degree of nodes) in Yellow River, Yangtze River, and Qiantang River estuaries regardless of cruises as well as in Pearl River estuary during the summer cruise, but even in Pearl River estuary during the winter cruise, *Nitrosopumilus* taxa with a relative abundance as low as 0.06% also had the highest connectivity (Fig. S6), indicating that AOA could hold a crucial position in the microbial networks across all the estuaries. Specifically, the *Nitrosopumilus* taxa commonly showed positive associations with heterotrophic bacteria affiliated to *Gammaproteobacteria* (primarily *Methylophilaceae*, *Halieaceae*, and *Comamonadaceae*), *Alphaproteobacteria* (primarily *Rhodobacteraceae*, *Sphingomonadaceae*, and SAR11 Clades), *Bacteroidota* (primarily *Flavobacteriaceae*), and *Actinobacteriota* (primarily *Sporichthyaceae* and *Ilumatobacteraceae*) (Figs. 5 and 6) and almost positive associations with eukaryotic phytoplankton affiliated to *Stramenopiles* and *Hacrobia* regardless of estuaries (Fig. 5). Previous studies also report strong associations between MGI taxa and heterotrophic gammaproteobacteria and alphaproteobacteria [23, 57, 69]. These consistent observations suggest that marine AOA as well-known autotrophs with possible heterotrophic potentials could have complex interactions with heterotrophic bacteria in processing organic matters via cross-feeding [70, 71], of which the processes and underlying mechanism are worthy of future investigations.

The genus *Poseidonia* displayed higher across-domain connectivity (degree of nodes ranging 2–43) than those of other *Poseidoniales* (MGII) genera in all the estuaries regardless of its relative abundance there (Fig. S6). This speculatively indicates that the occupy of ecological niches of *Poseidonia* could more rely on forming associations with bacteria than for other MGII genera, thus avoiding direct resource competition. In addition, the MGII genera *Poseidonia*, MGIIa-L1, MGIIb-O1, and/or MGIIb-O2 showed common associations with various bacterial taxa affiliated to *Gammaproteobacteria*, *Alphaproteobacteria*, *Bacteroidota*, and/or *Actinobacteriota* (Fig. 6). Many previous works have demonstrated various co-occurrence patterns of MGII archaea with (photo)heterotrophic bacteria affiliated to *Gammaproteobacteria* (such as SAR86 and *Oceanospirillales*), *Alphaproteobacteria* (such as *Rhodobacteraceae* and SAR11), and *Actinobacteriota* (such as *Actinomarinaceae*) [23, 69] in the marine waters less eutrophic than estuarine waters. In this study, we found expanded associations of MGII archaea with heterotrophic bacteria, likely due to higher amount and diversity of organic matters in the estuaries compared with oligotrophic waters [72, 73], which harbored more complex interactions of MGII archaea and heterotrophic bacteria with higher metabolic versatility of wider organic matter spectrum [74]. These findings indicate that future efforts should be made to unveil how dissolved organic matters mediate archaea-bacteria interactions. Despite above common patterns, the archaea-related microbial co-occurrences varied with estuaries, especially in the winter cruise, when Yellow River estuary had the simplest archaea-related network (with only 19 nodes and 10 edges; Table. S4), Yangtze River and Qiantang River estuaries showed similar archaea-related microbial co-occurrences, and Pearl River estuary possessed a unique pattern (Figs. 5 and 6). In the summer cruise, the archaea-related microbial co-occurrences showed more similarity among four estuaries with subtle distinctions. For example, archaea-related co-occurrences in Yellow River estuary were more complex than those in the winter cruise, but still simpler than those in the other estuaries (Fig. 5). Qiantang River estuary had the most complex archaea-related networks (with 515 nodes and 366 edges; Table. S4). These results suggest the extent of distinction of archaea-related microbial co-occurrences among the estuaries largely depended on geographic proximity and season. In this study, we found archaea-bacteria co-occurrences dominating the cross-domain associations in all the networks (ranging 90.0-98.1%). A previous study has highlighted the crucial role of planktonic bacteria-archaea co-occurrences on their biogeographic patterns [62]. Therefore, the distinct patterns of archaeal distribution across the estuaries could also be partly attributed to potential interactions between archaea and bacteria. Collectively, cross-domain co-occurrence patterns of archaea with other microbes (primarily bacteria) were contrasting across the estuaries, but many common associations were also detected along the geographic gradient. These findings suggest that potential cross-domain microbial interactions may contribute to shaping the biogeographic pattern of estuarine planktonic archaea, besides abiotic environmental and geographic factors, corresponding to our findings and discussion about the crucial role of bacterial community composition in shaping archaeal community above.

## Conclusion

This study demonstrated distinct patterns in archaeal community compositions, assembly mechanisms, and cross-domain co-occurrence in the surface waters in four major estuaries along the geographic gradient. The results showed that the relative abundance (in the prokaryotic community), taxonomic distribution, and community compositions of archaea drastically varied with estuary, season, sampling station (reflecting local environmental conditions), and their interactions to different degrees. This work also suggests that the cross-estuary dispersal of archaea was largely limited, and the assembly of archaea into prokaryotic communities within each estuary was likely governed by local environmental selection. Although the major roles of archaea in microbial networks across estuaries was peripherals and/or connectors, extensive and distinct cross-domain co-occurrence relationships of archaea with bacteria were found across the four estuaries, with AOA (primarily *Nitrosopumilus*) as the most crucial archaeal group. Furthermore, the expanded associations of MGII archaea with heterotrophic bacteria observed here could be the endogenous demand for co-processing high amount and diversity of organic matters in the estuarine ecosystem. This study performed a geographically-extensive and unified analysis of multiple estuaries to enhance our understanding of the common and differentiated characteristics of archaeal community in the estuarine ecosystem, providing new insights into roles of archaea in the microbial networks of this typical coastal ecosystem.

### Electronic supplementary material

Below is the link to the electronic supplementary material.

Supplementary Material 1

## Data Availability

The sequence data are available under accession number DRA016773 in the Sequence Read Archive of DDBJ (http://ddbj.nig.ac.jp/DRASearch).
