# Supplementary material for: Distinct patterns of distribution, community assembly and cross-domain co-occurrence of planktonic archaea in four major estuaries of China
Source: Environ Microbiome. 2023 Oct 7;18:75. doi: 10.1186/s40793-023-00530-9 (PMC10560434; doi:10.1186/s40793-023-00530-9)
Supplement: Supplementary file 1 — Supplementary Material 1 [file 40793_2023_530_MOESM1_ESM.pdf]

Supplementary information

Distinct patterns of distribution, community assembly and cross-domain co-occurrence of planktonic archaea in four major estuaries of China

Xuya Hu[1], Yujie Huang[1], Gaoke Gu[1], Hanjing Hu[1], Huizhen Yan[1], Huajun Zhang[1, 2, 3], Rui Zhang[4], Demin Zhang[1, 2, 3], Kai Wang[1, 2, 3*]

[1] State Key Laboratory for Managing Biotic and Chemical Threats to the Quality and Safety of Agro-products, School of Marine Sciences, Ningbo University, Ningbo, China

[2] Key Laboratory of Marine Biotechnology of Zhejiang Province, School of Marine Sciences, Ningbo University, Ningbo, China

[3] Collaborative Innovation Center for Zhejiang Marine High-Efficiency and Healthy Aquaculture, Ningbo, China

[4] Institute for Advanced Study, Shenzhen University, Shenzhen, China

[*] For correspondence. E-mail Kai Wang (wangkai@nbu.edu.cn)

Tel. 86-574-87600551; Fax 86-574-87608347.

The supplementary information contains:

- 6 Figures
- 4 Tables

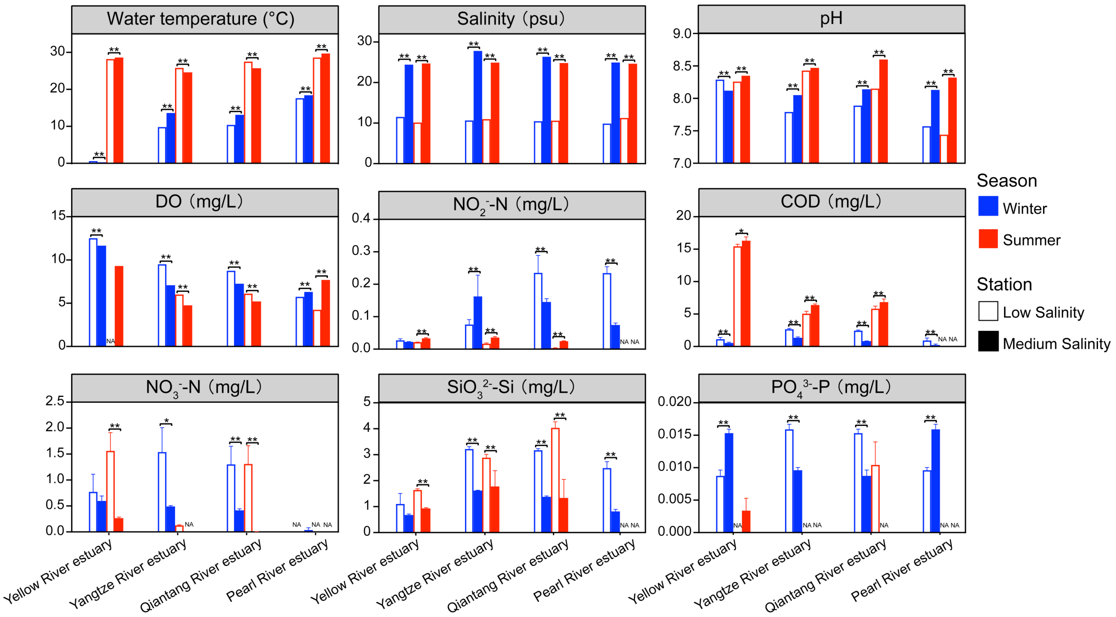

Figure S1. Dynamics of water environmental parameters across four estuaries during winter and summer cruises. Data present mean ± standard deviation (n = 5, except NO_3^- in the medium-salinity station of Qiantang River estuary (summer cruise) and Pearl River estuary (winter cruise) and PO_4^{3-} in the medium-salinity station of Yellow River estuary (summer cruise), for which n = 4). The significance of differences in environmental parameters between stations with low and medium salinity was tested using the Mann-Whitney *U* test ($^{*}P < 0.05$, $^{**}P < 0.01$) in each estuary during each seasonal cruise. DO, dissolved oxygen; NO_2^-, nitrite; COD, chemical oxygen demand; NO_3^-, nitrate; SiO_3^{2-}, silicate; PO_4^{3-}, phosphate. NA, not available.

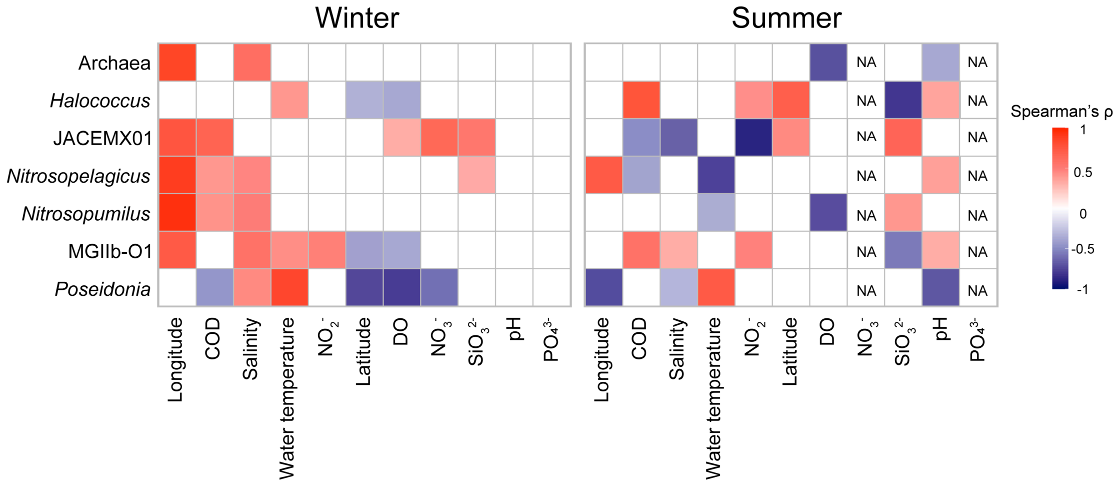

Figure S2. Heatmap showing Spearman's correlations of environmental and geographic factors with relative abundances of total archaea and major archaeal genera in prokaryotic communities during the winter or summer cruise. Colored cells indicate significance ($P < 0.05$). Cells with NA (not available) indicate that no calculations are performed when the coverage of environmental data less than 75% during the winter or summer cruise. COD, chemical oxygen demand; DO, dissolved oxygen.

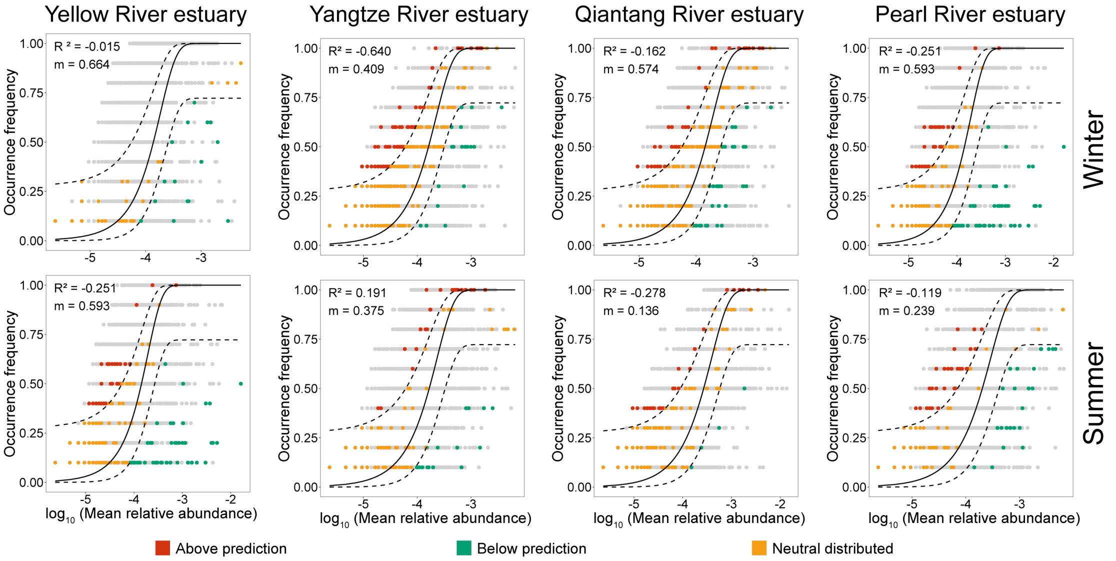

Figure S3. Fit of the neutral models for prokaryotic communities in each estuary during the winter or summer cruise with the metacommunity cross all four estuaries as the source. Archaeal ZOTUs that occurred more frequently than the model prediction are shown in red, while those occurred less frequently than predicted are shown in green. Dashed lines represent 95% confidence intervals around the model prediction and the archaeal ZOTUs fall within the confidence intervals are considered as neutrally distributed and shown in orange. Bacterial ZOTUs are shown in grey. R^2 values present the goodness of model fitting, ranging from 0 (no fit) to 1 (perfect fit).

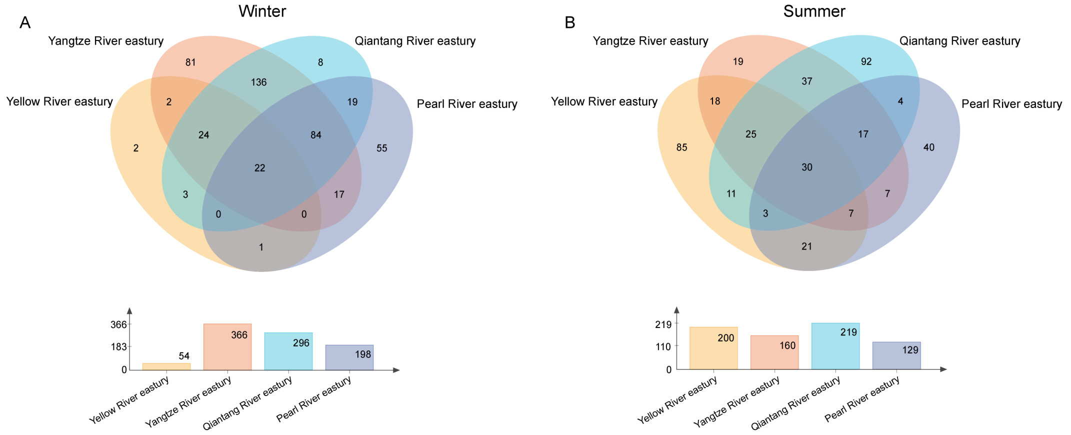

Figure S4. Venn diagrams showing the numbers of archaeal ZOTUs being unique and shared in four estuaries during winter (A) and summer cruises (B). The bar plots below the Venn diagrams present the number of archaeal ZOTUs in each estuary.

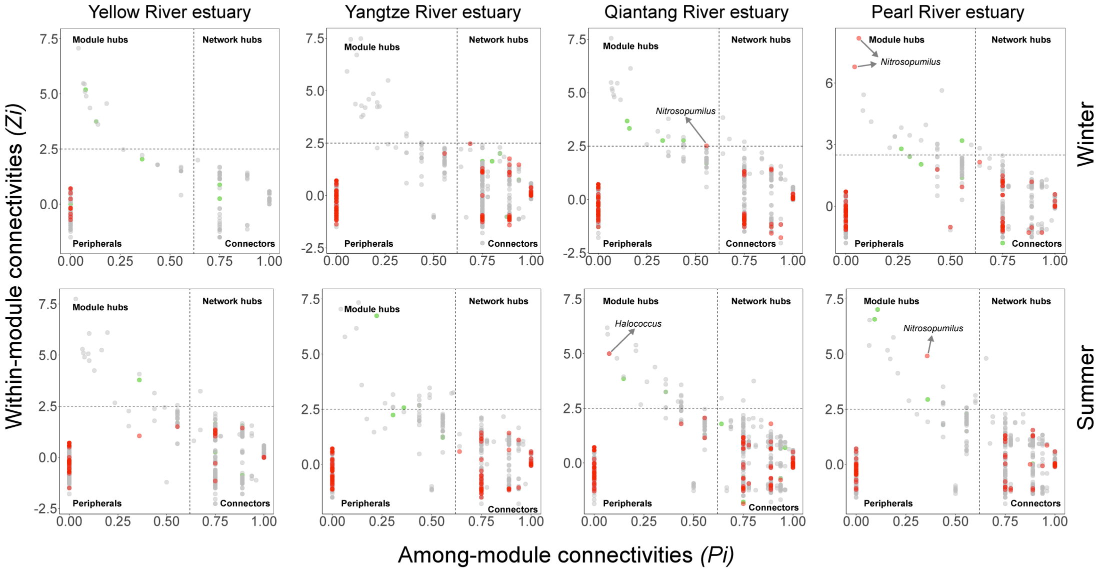

Figure S5. Classification of the nodes in the microbial co-occurrence networks. Four categories can be defined for each node: (1) peripheral nodes ($Zi \leq 2.5$, $Pi \leq 0.62$), (2) connectors ($Zi \leq 2.5$, $Pi > 0.62$), (3) module hubs ($Zi > 2.5$, $Pi \leq 0.62$), and (4) network hubs ($Zi > 2.5$, $Pi > 0.62$). Archaeal, bacterial, and phytoplanktonic ZOTUs are shown in red, grey, and green, respectively.

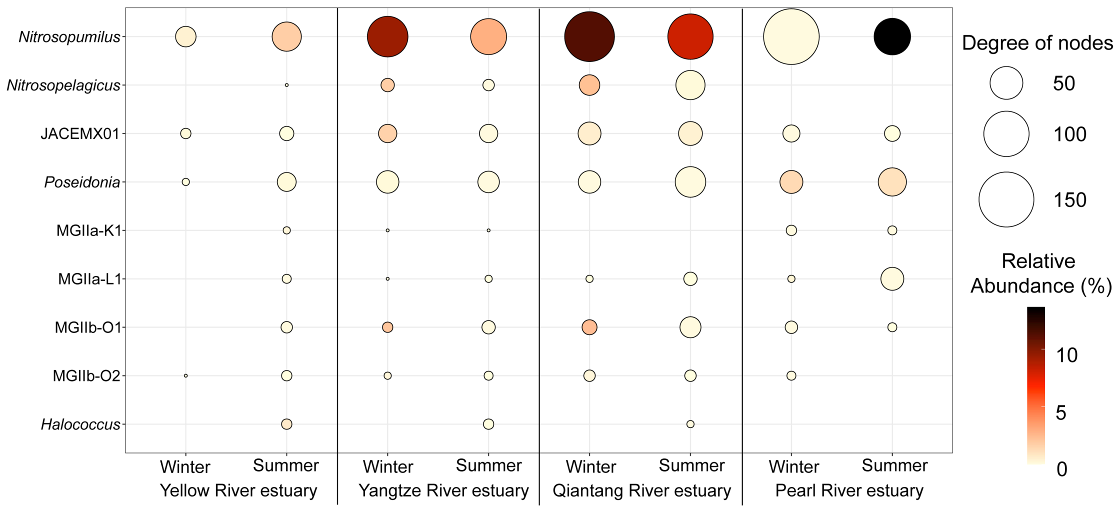

Figure S6. Bubble plot summarizing degree of nodes of cross-domain associations of major archaeal genera with their relative abundances in prokaryotic communities in each estuary during the winter and summer cruises. The size of bubbles indicates degree of nodes. The color of bubbles indicates the relative abundances of archaeal genera in prokaryotic communities.

Table S1. Three-way repeated measures analysis of variance (ANOVA) testing the influence of *Estuary*, *Season*, *Station*, and their interactions on relative abundances of total archaea and dominant archaeal taxa in prokaryotic communities.

		Total Archaea		*Halococcus*		JACEMX01		*Nitrosopelagicus*		*Nitrosopumilus*		MGIIb-O1		*Poseidonia*	
**Estuary**	**Factor**	F	*P*	F	*P*	F	*P*	F	*P*	F	*P*	F	*P*	F	*P*
All	*Estuary*	25.77	**< 0.001**	1.392	0.253	22.71	**< 0.001**	39.09	**< 0.001**	21.64	**< 0.001**	19.20	**< 0.001**	63.06	**< 0.001**
	Season	2.653	0.108	1.553	0.217	33.23	**< 0.001**	90.93	**< 0.001**	7.015	**0.010**	56.95	**< 0.001**	2.432	0.124
	Station	0.000	0.994	0.763	0.386	33.99	**< 0.001**	36.02	**< 0.001**	7.251	**0.009**	53.17	**< 0.001**	6.434	**0.014**
	Estuary × *Season*	38.19	**< 0.001**	1.402	0.250	20.12	**< 0.001**	31.34	**< 0.001**	34.64	**< 0.001**	18.97	**< 0.001**	3.613	**0.018**
	Estuary × *Station*	24.79	**< 0.001**	0.870	0.461	17.15	**< 0.001**	28.72	**< 0.001**	32.17	**< 0.001**	17.25	**< 0.001**	3.916	**0.012**
	Season × *Station*	98.46	**< 0.001**	0.774	0.382	10.73	**0.002**	36.60	**< 0.001**	86.59	**< 0.001**	51.46	**< 0.001**	61.68	**< 0.001**
	Estuary × *Season* × *Station*	19.41	**< 0.001**	0.866	0.463	20.55	**< 0.001**	33.87	**< 0.001**	19.42	**< 0.001**	16.65	**< 0.001**	46.73	**< 0.001**
Yellow River estuary	*Season*	12.12	**0.003**	1.439	0.248	9.654	**0.007**	8.203	**0.011**	0.000	1.000	24.25	**< 0.001**	18.68	**0.001**
	Station	5.473	**0.033**	0.842	0.372	2.980	0.104	0.068	0.798	0.000	1.000	11.80	**0.003**	0.202	0.659
	Season × *Station*	5.682	**0.030**	0.842	0.372	1.676	0.214	0.068	0.798	2.000	0.176	14.62	**0.001**	0.145	0.709
Yangtze River estuary	*Season*	36.00	**< 0.001**	7.714	**0.013**	30.85	**< 0.001**	56.15	**< 0.001**	23.56	**< 0.001**	25.91	**< 0.001**	5.166	**0.037**
	Station	7.288	**0.016**	0.857	0.368	26.60	**< 0.001**	0.807	0.382	14.57	**0.002**	26.50	**< 0.001**	6.333	**0.023**
	Season × *Station*	1.524	0.235	0.095	0.762	23.26	**< 0.001**	0.093	0.764	2.557	0.129	25.47	**< 0.001**	17.14	**0.001**
Qiantang River estuary	*Season*	10.61	**0.005**	2.068	0.170	2.032	0.173	41.86	**< 0.001**	0.444	0.515	30.81	**< 0.001**	1.793	0.199
	Station	10.27	**0.006**	2.068	0.170	6.522	**0.021**	43.68	**< 0.001**	0.927	0.350	26.00	**< 0.001**	2.069	0.170
	Season × *Station*	42.53	**< 0.001**	2.068	0.170	3.434	0.082	49.78	**< 0.001**	21.04	**< 0.001**	25.26	**< 0.001**	24.11	**< 0.001**
Pearl River estuary	*Season*	73.16	**< 0.001**	3.600	0.076	5.115	**0.038**	-	-	105.5	**< 0.001**	8.219	**0.011**	3.089	0.098
	Station	68.86	**< 0.001**	0.400	0.536	0.442	0.515	-	-	103.4	**< 0.001**	8.219	**0.011**	4.606	**0.048**
	Season × *Station*	114.5	**< 0.001**	0.400	0.536	1.434	0.249	-	-	104.6	**< 0.001**	7.253	**0.016**	51.72	**< 0.001**

Data in bold indicate significant influence ($P < 0.05$).

Table S2. Mantel tests demonstrating Spearman's correlations of the compositional variation of archaeal communities (based on Bray-Curtis dissimilarity) with variation in bacterial community composition (based on Bray-Curtis dissimilarity), environmental variables (based on Euclidean distance), and geographic distance (with 999 permutations) during the winter or summer cruise. Partial-Mantel tests demonstrating correlations between the compositional variation of archaeal communities and key significantly correlative factors (with Mantel ρ > 0.4) after controlled by each other. Three WYE_L samples, three WYE_M samples, and five WP_L samples of archaeal communities were excluded due to low read counts. Communities and environmental profiles with missing data were removed to generate a fully overlapping data set for each test.

Winter								Summer							
Variable	Mantel			Controlled by	Partial-Mantel			Variable	Mantel			Controlled by	Partial-Mantel		
	ρ	***P***	n		ρ	***P***	n		ρ	***P***	n		ρ	***P***	n
Bacteria	0.880	**0.001**	29	Geo.	0.731	**0.001**	29	Geo.	0.700	**0.001**	40	Bacteria	0.653	**0.001**	40
Bacteria	-	-	-	Temp.	0.637	**0.001**	29	Geo.	-	-	-	NO_2^-	0.782	**0.001**	30
Bacteria	-	-	-	DO	0.706	**0.001**	29	Bacteria	0.597	**0.001**	40	Geo.	0.526	**0.001**	40
Bacteria	-	-	-	COD	0.840	**0.001**	29	Bacteria	-	-	-	NO_2^-	0.799	**0.001**	30
Bacteria	-	-	-	SiO_3^{2-}	0.845	**0.001**	29	NO_2^-	0.499	**0.001**	30	Geo.	0.611	**0.001**	30
Bacteria	-	-	-	NO_3^-	0.839	**0.001**	28	NO_2^-	-	-	-	Bacteria	-0.046	0.754	30
Geo.	0.840	**0.001**	29	Bacteria	0.628	**0.001**	29	SiO_3^{2-}	0.305	0.002	30	-	-	-	-
Geo.	-	-	-	Temp.	0.525	**0.001**	29	Salinity	0.267	**0.001**	40	-	-	-	-
Geo.	-	-	-	DO	0.715	**0.001**	29	NO_3^-	0.135	0.018	24	-	-	-	-
Geo.	-	-	-	COD	0.809	**0.001**	29	pH	0.084	0.095	40	-	-	-	-
Geo.	-	-	-	SiO_3^{2-}	0.811	**0.001**	29	Temp.	0.070	0.067	40	-	-	-	-
Geo.	-	-	-	NO_3^-	0.823	**0.001**	28	DO	-0.002	0.529	35	-	-	-	-
Temp.	0.820	**0.001**	29	Bacteria	0.374	**0.001**	29	COD	-0.025	0.672	30	-	-	-	-
Temp.	-	-	-	Geo.	0.439	**0.001**	29	PO_4^{3-}	-0.362	0.968	9	-	-	-	-
Temp.	-	-	-	DO	0.499	**0.001**	29	-	-	-	-	-	-	-	-
Temp.	-	-	-	COD	0.786	**0.001**	29	-	-	-	-	-	-	-	-
Temp.	-	-	-	SiO_3^{2-}	0.768	**0.001**	29	-	-	-	-	-	-	-	-
Temp.	-	-	-	NO_3^-	0.817	**0.001**	28	-	-	-	-	-	-	-	-
DO	0.751	**0.001**	29	Bacteria	0.183	0.028	29	-	-	-	-	-	-	-	-
DO	-	-	-	Geo.	0.526	**0.001**	29	-	-	-	-	-	-	-	-
DO	-	-	-	Temp.	0.036	0.285	29	-	-	-	-	-	-	-	-
DO	-	-	-	COD	0.682	**0.001**	29	-	-	-	-	-	-	-	-
DO	-	-	-	SiO_3^{2-}	0.670	**0.001**	29	-	-	-	-	-	-	-	-
DO	-	-	-	NO_3^-	0.702	**0.001**	28	-	-	-	-	-	-	-	-

COD	0.484	**0.001**	29	Bacteria	-0.021	0.612	29	-	-	-	-	-	-	-	-
COD	-	-	-	Geo.	0.317	**0.001**	29	-	-	-	-	-	-	-	-
COD	-	-	-	Temp.	0.329	**0.003**	29	-	-	-	-	-	-	-	-
COD	-	-	-	DO	0.246	**0.002**	29	-	-	-	-	-	-	-	-
COD	-	-	-	SiO_3^{2-}	0.175	0.010	29	-	-	-	-	-	-	-	-
COD	-	-	-	NO_3^-	0.221	**0.003**	28	-	-	-	-	-	-	-	-
SiO_3^{2-}	0.462	**0.001**	29	Bacteria	-0.071	0.902	29	-	-	-	-	-	-	-	-
SiO_3^{2-}	-	-	-	Geo.	0.289	**0.003**	29	-	-	-	-	-	-	-	-
SiO_3^{2-}	-	-	-	Temp.	0.119	0.052	29	-	-	-	-	-	-	-	-
SiO_3^{2-}	-	-	-	DO	0.070	0.118	29	-	-	-	-	-	-	-	-
SiO_3^{2-}	-	-	-	COD	0.065	0.188	29	-	-	-	-	-	-	-	-
SiO_3^{2-}	-	-	-	NO_3^-	0.247	**0.003**	28	-	-	-	-	-	-	-	-
NO_3^-	0.461	**0.001**	28	Bacteria	0.118	0.102	28	-	-	-	-	-	-	-	-
NO_3^-	-	-	-	Geo.	0.380	**0.002**	28	-	-	-	-	-	-	-	-
NO_3^-	-	-	-	Temp.	0.439	**0.001**	28	-	-	-	-	-	-	-	-
NO_3^-	-	-	-	DO	0.290	0.009	28	-	-	-	-	-	-	-	-
NO_3^-	-	-	-	COD	0.210	0.016	28	-	-	-	-	-	-	-	-
NO_3^-	-	-	-	SiO_3^{2-}	0.253	0.007	28	-	-	-	-	-	-	-	-
pH	0.351	**0.001**	29	-	-	-	-	-	-	-	-	-	-	-	-
Salinity	0.341	**0.001**	29	-	-	-	-	-	-	-	-	-	-	-	-
PO_4^{3-}	0.143	0.017	29	-	-	-	-	-	-	-	-	-	-	-	-
NO_2^-	0.111	0.079	29	-	-	-	-	-	-	-	-	-	-	-	-

Data in bold indicate significant correlations ($P < 0.05$). Bacteria, Bacterial community composition; Geo., geographic distance; Temp., water temperature; DO, dissolved oxygen; COD, chemical oxygen demand.

Table S3. Mantel tests demonstrating Spearman's correlations of the compositional variation of bacterial communities (based on Bray-Curtis dissimilarity) with variation in archaeal community composition (based on Bray-Curtis dissimilarity), environmental variables (based on Euclidean distance), and geographic distance (with 999 permutations) during the winter or summer cruise. Partial-Mantel tests demonstrating correlations between the compositional variation of bacterial communities and key significantly correlative factors (with Mantel ρ > 0.4) after controlled by each other. Three WYE_L samples, three WYE_M samples, and five WP_L samples of archaeal communities were excluded due to low read counts. Communities and environmental profiles with missing data were removed to generate a fully overlapping data set for each test.

Winter								Summer							
Variable	Mantel			Controlled by	Partial-Mantel			Variable	Mantel			Controlled by	Partial-Mantel		
	ρ	***P***	n		ρ	***P***	n		ρ	***P***	n		ρ	***P***	n
Archaea	0.880	**0.001**	29	Temp.	0.637	**0.001**	29	Archaea	0.597	**0.001**	40	NO_2^-	0.799	**0.001**	30
Archaea	-	-	-	DO	0.706	**0.001**	29	Archaea	-	-	-	Salinity	0.530	**0.001**	40
Archaea	-	-	-	Geo.	0.731	**0.001**	29	Archaea	-	-	-	SiO_3^{2-}	0.795	**0.001**	30
Temp.	0.749	**0.001**	40	Archaea	0.344	**0.001**	29	NO_2^-	0.572	**0.001**	30	Archaea	0.412	**0.001**	30
Temp.	-	-	-	DO	0.316	**0.001**	40	NO_2^-	-	-	-	Salinity	0.471	**0.001**	30
Temp.	-	-	-	Geo.	0.455	**0.001**	40	NO_2^-	-	-	-	SiO_3^{2-}	0.396	**0.001**	30
DO	0.719	**0.001**	40	Archaea	0.424	**0.001**	29	Salinity	0.509	**0.001**	40	Archaea	0.550	**0.001**	40
DO	-	-	-	Temp.	0.093	0.038	40	Salinity	-	-	-	NO_2^-	0.429	0.002	30
DO	-	-	-	Geo.	0.450	**0.001**	40	Salinity	-	-	-	SiO_3^{2-}	0.568	**0.001**	30
Geo.	0.717	**0.001**	40	Archaea	-0.077	0.889	29	SiO_3^{2-}	0.429	**0.001**	30	Archaea	0.298	**0.001**	30
Geo.	-	-	-	Temp.	0.350	**0.001**	40	SiO_3^{2-}	-	-	-	NO_2^-	0.299	**0.001**	30
Geo.	-	-	-	DO	0.446	**0.001**	40	SiO_3^{2-}	-	-	-	Salinity	0.549	**0.001**	30
pH	0.375	**0.001**	40	-	-	-	-	pH	0.366	**0.001**	40	-	-	-	-
SiO_3^{2-}	0.371	**0.001**	40	-	-	-	-	Geo.	0.350	**0.001**	40	-	-	-	-
COD	0.344	**0.001**	40	-	-	-	-	NO_3^-	-0.066	0.858	24	-	-	-	-
NO_3^-	0.319	**0.001**	34	-	-	-	-	DO	-0.074	0.927	35	-	-	-	-
Salinity	0.244	**0.001**	40	-	-	-	-	Temp.	-0.076	0.964	40	-	-	-	-
NO_2^-	0.199	**0.003**	40	-	-	-	-	COD	-0.116	0.968	30	-	-	-	-
PO_4^{3-}	-0.028	0.779	40	-	-	-	-	PO_4^{3-}	-0.425	0.978	9	-	-	-	-

Data in bold indicate significant correlations ($P < 0.05$). Archaea, Archaeal community composition; Temp., water temperature; DO, dissolved oxygen; Geo., geographic distance; COD, chemical oxygen demand.

Table S4. Summary of the features of microbial co-occurrence networks detected by FlashWeave. Topological features were calculated after removing indirect and environment-related edges. Archaeal-related topological features were calculated for archaeal co-occurrence networks. Only direct edges (with percentage in all edges in parentheses) were visualized in the chord diagrams (Fig. 5). Archaeal nodes (with percentage in all microbial nodes in parentheses), archaeal edges (with percentage in all microbial edges in parentheses), and positive edges (with percentage in all archaeal edges in parentheses) were shown.

Estuary	Season	Raw ZOTUs		ZOTUs selected for network calculations (Reads ≥ 100, Sample coverage ≥ 15%)		FlashWeave Result			Archaeal-related topological features							
		**ZOTUs**	**Reads**	**ZOTUs**	**Reads (%)**	**Variables**	**indirect & direct edges**	**direct edges (%)**	**Nodes (%)**	**Edges (%)**	**Positive edges (%)**	**Modularity**	**Clustering coefficient**	**Network diameter**	**Average path length**	**Average degree**
Yellow River estuary	Winter	4679	196991	1211	128190 (65.07%)	1214	7514	595 (7.91%)	19 (2.50%)	10 (1.69%)	8 (80.00%)	1.018	0	2	1.091	1.053
	Summer	7908	414878	2293	333434 (80.37%)	2296	48272	1567 (3.24%)	110 (5.74%)	68 (4.35%)	57 (83.82%)	1.173	0	4	1.441	1.236
Yangtze River estuary	Winter	9459	175969	2056	116284 (66.08%)	2059	53209	1604 (3.01%)	229 (12.92%)	164 (10.26%)	117 (71.34%)	1.539	0.034	10	2.895	1.432
	Summer	76 55	441389	2415	400187 (90.67%)	2418	77720	1733 (2.22%)	218 (10.85%)	150 (8.68%)	118 (78.67%)	1.282	0.083	6	1.68	1.376
Qiantang River estuary	Winter	9328	189518	2040	137465 (72.53%)	2043	36964	1499 (4.05%)	215 (11.92%)	144 (9.63%)	109 (75.69%)	1.412	0	5	1.743	1.340
	Summer	8840	441814	2239	358172 (81.07%)	2242	213318	2270 (1.06%)	300 (14.19%)	222 (9.8%)	184 (82.88%)	1.203	0.037	7	1.988	1.480
Pearl River estuary	Winter	6882	213057	2221	167453 (78.60%)	2224	68600	1692 (2.46%)	275 (14.55%)	218 (12.93%)	207 (94.95%)	0.897	0	6	1.944	1.585
	Summer	6528	436220	2250	389455 (89.28%)	2253	210171	1960 (0.93%)	178 (8.64%)	132 (6.76%)	102 (77.27%)	1.276	0.076	6	1.844	1.483
